# Revisiting the effective connectivity within the distributed cortical network for face perception

**DOI:** 10.1016/j.ynirp.2021.100045

**Published:** 2021-08-17

**Authors:** Roman Kessler, Kristin M. Rusch, Kim C. Wende, Verena Schuster, Andreas Jansen

**Affiliations:** a Laboratory for Multimodal Neuroimaging, Department of Psychiatry and Psychotherapy, University of Marburg, Germany; b Center for Mind, Brain and Behavior, University of Marburg and University of Giessen, Germany; c Norwegian University of Science and Technology (NTNU), Gjøvik, Norway; d University of Applied Sciences, Darmstadt, Germany; e Department of Neurology and Neurorehabilitation, Hospital zum Heiligen Geist, Academic Teaching Hospital of the Heinrich-Heine-University Düsseldorf, Kempen, Germany; f Institute of Medical Psychology and Medical Sociology, Faculty of Medicine, University of Freiburg, Germany; g The Neuro (Montréal Neurological Institute-Hospital), McGill University, Montréal, Canada; h Core-Unit Brainimaging, Faculty of Medicine, University of Marburg, Germany

**Keywords:** Conceptual replication, Dynamic causal modeling, Emotion processing, Face perception, fMRI

## Abstract

The classical core system of face perception consists of the occipital face area (OFA), fusiform face area (FFA), and posterior superior temporal sulcus (STS). The functional interaction within this network, more specifically the effective connectivity, was first described by Fairhall and Ishai (2007) using functional magnetic resonance imaging and dynamic causal modeling. They proposed that the core system is hierarchically organized; information is processed in a parallel and predominantly feed-forward fashion from the OFA to downstream regions such as the FFA and STS, with no lateral connectivity, i.e., no connectivity between the two downstream regions (FFA and STS). Over a decade later, we conducted a conceptual replication of their model using four different functional magnetic resonance imaging data sets. The effective connectivity within the core system was assessed with contemporary versions of dynamic causal modeling.

The resulting model of the core system of face perception was densely interconnected. Using hierarchical linear modeling, we identified several significant forward, backward, and lateral connections in the core system of face perception across the data sets. Face perception increased the forward connectivity from the OFA to the FFA and OFA to the STS and increased the inhibitory backward connectivity from the FFA to the OFA, as well as the lateral connectivity between the FFA and STS. Emotion perception increased forward connectivity between the OFA and STS and decreased the lateral connectivity between the FFA and STS. Face familiarity did not significantly alter these connections.

Our results revise the 2007 model of the core system of face perception. We discuss the potential meaning of the resulting model parameters and propose that our revised model is a suitable working model for further studies assessing the functional interaction within the core system of face perception. Our work further emphasizes the general importance of conceptual replications.

## Highlights

• We revised an early connectivity model of face perception using multiple data sets.
• Connectivity estimates were highly similar across different data sets.
• The core system of face perception is highly interconnected.

## 1 Introduction

Face processing is mediated by a widely distributed neural network. This network is often divided into a core system and an extended system (Haxby model, Haxby et al., 2000). The core system is involved in the processing of basic information about faces. It consists of several bilateral brain regions in the occipitotemporal cortex; specifically, the occipital face area (OFA) in the inferior occipital gyrus, the fusiform face area (FFA) in the middle fusiform gyrus, and an area in the posterior superior temporal sulcus (STS). According to the Haxby model, the OFA is responsible for the early processing of physical features of face stimuli and sends its output to both the FFA and STS. The FFA is associated with the representation of invariant aspects of the face (e.g., face identity), while the STS processes changeable aspects of facial expression (e.g., lip movements and the direction of eye-gaze). Beyond the core system, there are several additional regions that contribute to face perception, such as the amygdala, insula, inferior frontal gyrus, and orbitofrontal cortex (Gobbini and Haxby, 2007). This extended system tends to be task-specific and comes into play if additional information is extracted from faces, such as attractiveness or biographical information.

Only a few studies have previously investigated the assumptions made by the Haxby model with respect to the interplay between the face-sensitive regions. An understanding of the interaction between these areas, however, is crucial for unraveling how the human brain processes faces and might also provide new insights into the pathophysiology of disorders where face perception is impaired (e.g., prosopagnosia). One method to test the interactions between brain regions is dynamic causal modeling (DCM) (Friston et al., 2003). DCM is used to test hypotheses about the neural network structure. It estimates the directed coupling between brain areas (effective connectivity) and the changes in coupling caused by experimental manipulations (i.e., context). A few different neural network models (i.e., DCMs) have been developed for the face perception system. These DCMs assessed the neural dynamics within the core system of face perception, the interaction between the core system and extended system, and the effects of ‘emotions’ and ‘fame’ on the effective connectivity within those networks (e.g., Dima et al., 2011; Fairhall and Ishai, 2007; Furl, 2015; Furl et al., 2015; Herrington et al., 2011). They were typically limited to one hemisphere but have recently been expanded by bilateral DCMs, including interactions between both hemispheres (Frässle et al, 2016b, 2016c, 2016b).

**Fig. 1:**
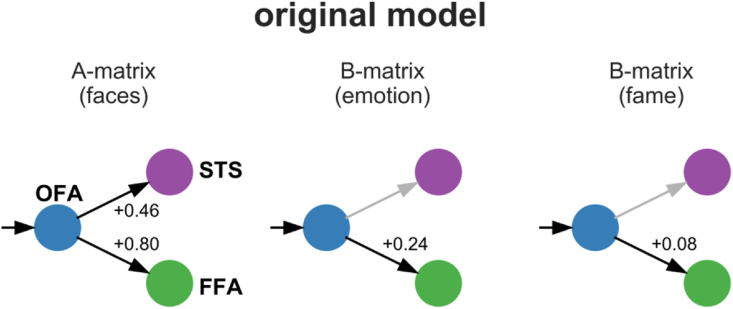
Dynamic causal model of the interactions within the core system of face perception by Fairhall and Ishai (2007). Driving input (faces) enters the OFA, which propagates the information in a parallel manner toward the FFA and STS. Assumptions about the effect of faces were drawn from the A-matrix. Assumptions about the effects of emotion and fame were drawn from separate B-matrices (see Material and Methods for further information on the terminology of DCM, see Discussion for further information on the modeling strategy).

The first study that used DCM to describe the interactions between face-sensitive brain regions was published almost 15 years ago. In this study, Fairhall and Ishai (2007) tested DCMs, which were built based on the Haxby model and described the interactions within the core system. Not only did they show how the OFA, FFA, and STS interact during face processing, but they also assessed how factors like emotional valence and the fame of faces influenced those interactions (see Fig. 1 for a graphical depiction of their model). Their study's main results were:

i. The OFA propagates face-specific content simultaneously to the FFA and STS in a feed-forward fashion.
ii. Backward connections to the OFA and collateral connections between the FFA and STS were not present in their proposed model.
iii. Emotional valence enhanced connectivity from the OFA to the FFA.
iv. ‘Fame’ enhanced connectivity from the OFA to the FFA.

The Fairhall and Ishai (2007) study has been highly influential and widely cited since it was published, and it further makes far-reaching claims on how the brain regions in the core system interact during face processing and how these interactions are modulated. Various studies investigating the connectivity within the core system of face perception have been published, building upon these results (Elbich et al., 2019; Frässle et al., 2016a, 2016b, 2016c, 2016b; He et al., 2015; Lohse et al., 2016; Nagy et al., 2012; Nguyen et al., 2014; Sato et al., 2017). However, the study's results have never been formally replicated, neither in different samples nor with different strategies of analysis. Therefore, the aim of the present study was to investigate the degree to which we can reproduce the results from the study by Fairhall and Ishai (2007).

Concerns about the reproducibility of neuroimaging findings have been steadily raised in recent years since numerous studies have shown that the results of previous experiments could not be replicated (Gorgolewski and Poldrack, 2016). One reason for this is that results obtained can be highly dependent on the tools being used as well as differences in the experimental setup, pipeline, or statistical methods (Bedenbender et al., 2011; Botvinik-Nezer et al., 2020; Weissenbacher et al., 2009). Reproducibility can be assessed with different approaches (Diener and Biswas-Diener, 2016). An exact replication can be performed by attempting to repeat the original study in the best way possible, i.e., using identical paradigms and tools for analysis. However, there is also the option of a conceptual replication, wherein the researchers are not interested in simply repeating the steps of the original study in an exact and sequential manner. Instead, they may be interested in answering the very same research question as that in the original study by using tools that are similarly suitable to find those answers. Both types of replications are important since they each give us new but complementary information. While exact replications strengthen our belief in the findings from the original research, conceptual replications can strengthen the theoretical idea behind the findings. In other words, conceptual replications offer insights into how generalizable the findings are. In the present study, we aimed to conduct a conceptual replication of the core results of the study by Fairhall and Ishai (2007). We were not interested in whether these results could be reproduced in one specific sample, with one specific face perception task, and with one specific analysis pipeline. Rather, we aimed to assess whether the findings can be replicated over several samples, different implementations of face processing tasks, and different analysis methods.

In summary, we investigated face-specific interactions in the core system, i.e., between the OFA, FFA, and STS in the right hemisphere. We expected similar results to those from the study conducted by Fairhall & Ishai (Fairhall and Ishai, 2007), namely an increase in forward connectivity from the OFA to the FFA and from the OFA to the STS. Furthermore, we investigated the influence of ‘emotion’ and ‘fame’ on the strength of the connections between brain regions of the core system. We expected ‘emotion’ and ‘fame’ to increase the connectivity from the OFA to the FFA, similar to what was observed in the original study. By analyzing four different samples, three of which were acquired in our laboratory, we aimed to increase the generalizability of our results. In all four samples, the processing of faces was investigated; emotion processing was additionally assessed in two samples. The fourth sample, which was retrieved from an open neuroimaging platform (Wakeman and Henson, 2015), allowed us to investigate the effect of ‘fame’. All the studies from which the samples were obtained used distinct paradigms and participants. To combine our results with these studies, we applied a hierarchical linear modeling (HLM) approach.

## 2 Material and Methods

### 2.1 Study samples

We analyzed four samples of healthy participants (referred to in the manuscript as data sets A–D, studies A–D, paradigms A–D, samples A–D, and so forth). Three of these data sets (A, B, and C) were retrieved from ongoing (and therefore yet unpublished) studies in our lab (Laboratory for Multimodal Neuroimaging, Department of Psychiatry, University of Marburg, Germany). Studies A and B were originally planned to investigate the changes in connectivity in the face perception network associated with facial emotion processing. Study C initially assessed the impact of female hormones on brain structure and function, and, on the face-processing network. Written informed consent was provided by all the participants. The fourth data set (data set D) was obtained from the OpenNeuro project (openneuro.org), accession number ds000117, Wakeman and Henson (2015). In Table 1, we summarized detailed information on the participants' characteristics of studies A-D and the original study (Fairhall and Ishai, 2007) (henceforth referred to as ‘study FI’). Participants in samples A and B were investigated just once. Participants in sample C (all female) were investigated twice, with 1–25 weeks between sessions (mean = 7 weeks); one measurement took place during the mid-luteal phase and the other during the early follicular phase of the menstrual cycle. Participants in sample D were measured ten times each with the same face perception paradigm. Sessions in which there was no significant activation in each of the three regions of the core system were excluded from DCM analyses (see chapter 2.4.2[i]). Therefore, we report both the total number of participants and sessions for each study (rows 1 and 2) and the participants and sessions included in the final analyses (remaining rows). In study FI, participants were measured five times with four different paradigms (Fairhall and Ishai, 2007).

**Table 1:** Sample characteristics of each study.

Sample	*A*	*B*	*C*	*D*	*FI*
total number of participants	25	31	20	16	n.a.
total number of sessions per participant	1	1	2	10	5
number of participants included	23	27	17	16	10
number of sessions included per participant	1	1	1–2	5-10 (Md: 8)	5
number of males	11	13	0	9	5
number of females	12	14	17	7	5
age (years)	24 (Md)	24 (Md)	24 (Md)	n.a.	25 (mean)
minimum age (years)	21	20	20	23*	n.a.
maximum age (years)	29	29	28	37*	n.a.

Abbreviations: Md, median; n.a., Information not available. *Study D: The age range of all 19 participants was included in the online repository. However, at the time of our analysis, the data for only 16 participants were accessible. Therefore, the age range in study D might differ from that shown above.

### 2.2 Functional paradigms

**Fig. 2:**
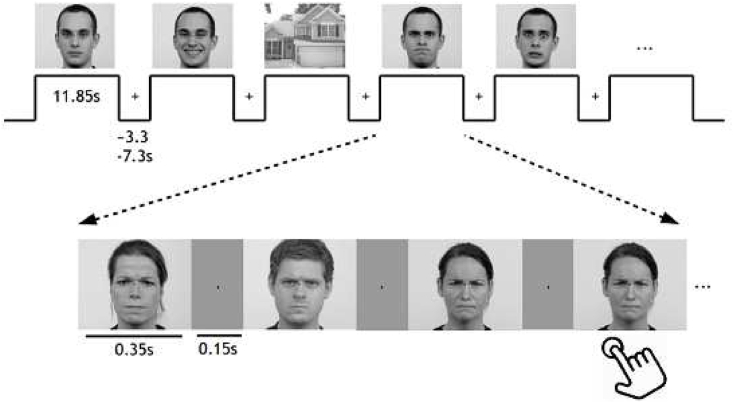
Experimental paradigm for study A. In paradigm A, pictures of either neutral, happy, angry, or fearful faces (Langner et al., 2010) were shown in the experimental condition, and houses were shown in the control condition. Single stimuli and blocks were intervened by a gray screen. Participants were instructed to maintain the fixation of their gaze throughout the entire experiment. They were further instructed to press a button if a stimulus was presented twice in a row (one to two times per block). The total experiment lasted about 30 min.

The paradigms of all the data sets were constructed to tackle questions related to face perception. Participants viewed face stimuli in the experimental conditions and non-face stimuli (i.e., houses or phase-scrambled images) in the control conditions. Studies A–D used photographs of faces, while study FI used different face stimuli (line drawings of faces, famous faces, emotional faces, and unfamiliar faces). Paradigms A–C were set up in a block design similar to study FI, whereas paradigm D used an event-related design. All the paradigms included a simple task, such as a one-back task (paradigm A–C) or symmetry rating task (paradigm D). Study FI did not include any accompanying task. We have presented paradigm A in Fig. 2. More detailed descriptions of paradigms A–C can be found in the supplementary methods. A description of paradigm D is found in the study by Wakeman and Henson (2015). Paradigm FI is described in study FI (Fairhall and Ishai, 2007).

One crucial difference between the paradigms was the inclusion of emotional or famous faces. Paradigm A used four different emotional expressions, namely neutral, fearful, happy, and angry, separated into different blocks (Fig. 2). Paradigm B used two different emotional expressions, neutral and fearful. Paradigms C and D used neutral faces instead of particularly emotional expressions. Paradigms A–C used non-famous faces, whereas paradigm D used non-famous as well as famous faces.

### 2.3 Data acquisition

High resolution structural images and blood oxygen level-dependent functional images of all four data sets were acquired using Siemens 3T TIM TRIO MR scanners (Siemens, Erlangen, Germany). Study FI used a 3T Philips Intera scanner (Philips, Hamburg, Germany). All measurement volumes for the functional image acquisitions covered the entire core system of face perception. Information on the properties of the scanning sequences is detailed in the supplementary methods.

### 2.4 Data analysis

#### 2.4.1 Preprocessing and statistical analysis of brain activity

Analyses of the magnetic resonance imaging (MRI) data sets A, B, and C were conducted using Statistical Parametric Mapping 12 (SPM12) (https://www.fil.ion.ucl.ac.uk/spm/). Data set D was processed using FSL (http://fsl.fmrib.ox.ac.uk/fsl/fslwiki/), and study FI used SPM5. In all the data sets, preprocessing included motion correction, spatial normalization (except study A), and spatial smoothing. Statistical analyses were conducted using a general linear model. We modeled ‘faces’ as regressors of interest, and the control condition (e.g., houses or scrambled faces) were modeled as separate regressors, following which we contrasted the ‘face’ vs. control conditions. Here, we did not differentiate between neutral, emotional, or famous faces. Similarly, we did not differentiate between the different control conditions. Nuisance regressors included the six realignment parameters. A more detailed description of the specific analysis pipelines can be found in the supplementary methods. Notably, we could have used the raw data of each data set and implemented an identical preprocessing pipeline for all paradigms. However, to increase the generalizability, we decided to use the preprocessed data sets. All the procedures that were implemented by the respective authors represent valid implementations of preprocessing pipelines.

#### 2.4.2 Dynamic causal modeling

The connectivity pattern of the core system of face perception was assessed with DCM (Friston et al., 2003; Zeidman et al., 2019a). DCM is a framework to disentangle effective connectivity in neuroimaging data. In its original formulation, it models the brain as a deterministic input-output system using the following differential equation:

$$\frac{dz}{dt}=\left(A+\overset{m}{\underset{j=1}{\sum }}u_{j}B^{\left(j\right)}\right)z+Cu$$

In this equation, z depicts the neural activations, u is the experimental input or context, A describes the endogenous connection strengths, $B^{\left(j\right)}$ models how the experimental context $u_{j}$ affects connectivity in the network, and C models how the experimental input directly influences the neural activity within the regions of interest. The dynamics of the neural activations are translated into predictions about the blood oxygen level-dependent signal by a hemodynamic forward model (Buxton et al., 1998). The model parameters are then estimated by maximizing the negative free energy.

DCM enables inferences at different levels, such as the inferences on model space and parameter space of any given model. In the following sections, we will describe (i) the extraction of time series from the OFA, FFA, and STS, (ii) the specification of the model space, and (iii) the specific DCM analyses assessing the network parameters within and across the studies.

(i) Identification of the OFA, FFA, and STS

DCMs were constructed for the core system of face perception within the right hemisphere (OFA, FFA, and STS). In the following paragraphs, we describe how we defined regions of the core system and extracted the time series of the respective regions.

Two different approaches were used to identify brain regions at the single-participant level. Regarding the choice of the preprocessing steps, we did not adopt one specific standard for the present study. Instead, to increase the generalizability, we applied the approaches for time series extraction that had been used by the authors in the respective studies. The first approach was used for data set A, in which the MRI data was not normalized. In this data set, we manually identified the peak activation clusters at a single participant level in the native image space (Frässle et al., 2016c). We superimposed the participants' co-registered structural image with the t-map for the contrast “faces > control condition.” We then identified the OFA, FFA, and STS as the clusters with the highest activities in the inferior occipital gyrus, posterior fusiform gyrus, and the posterior superior temporal sulcus, respectively. If several clusters were candidates for a particular region, we used the activation strength and symmetry to an analog cluster in the opposite hemisphere as criteria. The second approach was used for data sets B, C, and D, in which the MRI data was normalized (Kessler et al., 2020; Sladky et al., 2015). For each study, we first assessed the brain activity at the group level. The individual contrast images (“faces > control condition”) were entered in a random-effects analysis using a one-sample *t*-test. We identified the group peak activation coordinates for the OFA, FFA, and STS using the same anatomical criteria as described above. Next, we identified participant-specific peak coordinates for these regions. A peak coordinate was defined in each participant as the voxel with the highest t-value within a mask (radius, 12 mm) centered on the group peak coordinate for the respective region.

For all the data sets, the time series were extracted for each region and participant/session as the first principal component of all the voxels activated at a threshold of 0.001, uncorrected for multiple comparisons, located within a radius of 4 mm around the participant-specific peak voxel. Due to the lower overall activation in data set D, we increased the statistical threshold to 0.1 (uncorrected) for this data set. Participants/sessions in which no activity was found at the pre-defined statistical threshold in at least one region were excluded from further analyses. Two participants from data set A, four from data set B, and three from data set C were excluded (Table 1).

(ii) Specification of model space

**Fig. 3:**
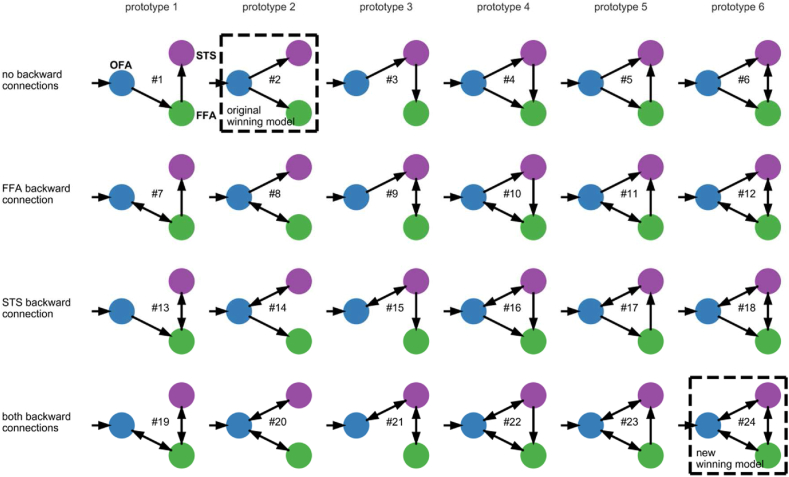
**Model space.** Models of the core system of face perception tested with Bayesian model selection (BMS). Connectivity was investigated by modifying the forward, lateral, and feedback connections between the three investigated regions, namely the OFA (blue), FFA (green), and STS (purple). Driving input by faces was set on the OFA (C-matrix, short arrow). All context-independent connections (A-matrix) are displayed with arrows, except the inhibitory self-connections. All interregional connections were modulated (B-matrix) by ‘faces’ (studies A-D), ‘emotion’ (studies A and B), and ‘fame’ (study D). The winning model of the original study FI (#2) and the winning model of our revised model comparisons (#24, see Results section) are marked with dashed rectangles. (For interpretation of the references to color in this figure legend, the reader is referred to the Web version of this article.)

For all the data sets, we specified models similar to those in study FI (Fairhall and Ishai, 2007). All the models consisted of three regions: the OFA, FFA, and STS. These regions were interconnected differently, varying in the presence or absence of context-independent connections (A-matrix). In total, we constructed 24 models (Fig. 3). A ‘face’ input regressor was set onto the OFA in all the models (C-matrix). Furthermore, we allowed ‘faces’ to modulate all available interregional connections within each model (B-matrix). Intra-regional connections (i.e., self-connections) were not modulated in the B-matrix. Our model specification was informed by the models of study FI, as well as by assuming the OFA as an input region and by allowing an input to be distributed to all downstream regions by at least one possible route. However, our model specification deliberately differed from that in study FI with regard to the specification of the influence of face perception. In study FI, the influence of the presentation of faces, in comparison to other objects, was not modeled explicitly (see Section 4.2 for a detailed discussion). To assess the effects of ‘emotion’ and ‘fame,’ we further allowed the modulation of all interregional connections by ‘emotion’ (data set A and B) and ‘fame’ (data set D). At this point, we decided again to use a different modeling procedure compared to study FI (see discussion 4.2. for a more detailed explanation).

Whereas study B comprised only one emotion (fear, plus neutral expression), the regressor for ‘emotion’ was interpreted in a straightforward manner (i.e., as an effect of ‘fear’). In study A, however, three different expressions (happiness, fear, anger) were presented alongside neutral facial expressions. We deliberately pooled across all emotional expressions, except ‘neutral,’ to construct a regressor for ‘emotion’ to stay consistent with the approach of the original study (Fairhall and Ishai, 2007). In that study, pooling was conducted across two emotions; specifically, fear and happiness. We acknowledge that different emotions may lead to different activity and connectivity.

(iii) DCM Analysis

Our DCM analysis can be divided into three steps. First, we conducted Bayesian model selection (BMS) to assess which model is best supported by the data separately for each participant and study. Second, we used Bayesian model averaging (BMA) to estimate averaged model parameters separately for each participant and study. Last, as the main aim of the present study, we used HLM to assess model parameters across the participants and studies.

***Bayesian model selection****:* First, we compared the different models using random-effects BMS separately for each study (Stephan et al, 2009, 2010). We quantified the models’ goodness-of-fit based on the negative free energy, an approximation to the log model evidence (Friston et al., 2007). As a result of BMS, we obtained the posterior and exceedance probabilities for each model, assessed across all the participants within each study. Our objective was not just to assess whether the winning models in our data sets were congruent with the winning model reported in study FI but also to qualitatively assess if the winning model is consistent across all the studies.

***Bayesian model averaging***: Next, we calculated the averaged model parameters via BMA (Penny, 2012; Penny et al., 2010)). BMA uses the posterior model probabilities of all the models of a particular participant and calculates a weighted average model. The weights were determined by the respective posterior model probabilities. BMA, therefore, accounts for the uncertainty of each model (Stephan et al., 2010). The results are presented at the single participant and group levels. The single participant results allow the visualization of the variance across the participants within one study (see Figs. S1–S4). The group results allow the description of the variability of the results across the studies. Two-sided one-sample t-tests were conducted for each connection per study to assess whether a connection parameter significantly differed from zero. We applied a Bonferroni family-wise error correction within each matrix for a particular study, resulting in a threshold of $\alpha_{Bonf}=\frac{\alpha }{n}=\frac{0.05}{6}$, with n as the number of tests, and α as the native false-positive threshold. We tested inter-regional connections (i.e., off-diagonal elements of the respective matrix). Self-connections were first converted to unit Hertz by applying $a_{Hz}=\;-0.5*e^{\left\{a_{logscale}\right\}}$ to be on the same scale as the inter-regional connections (Zeidman et al., 2019a). We did not test self-connections for significance because those are negative by definition (Fig. S2).

Studies C and D included more than one experimental session per participant. In study C, each participant was measured twice, with the participants’ hormone levels differing between the two experimental sessions. Therefore, we have reported the BMS and BMA results for both sessions separately. In study D, we included five to nine experimental sessions per participant depending on the number of sessions in which all the regions could be clearly identified (see 2.4.2.[i]). The division into two separate sessions was not motivated by an experimental manipulation as in study C. For the sake of clarity, we will not report group-BMS and group-BMA results across all nine sessions in study D. However, for the subsequent analysis with HLM, we included each participant and session appropriately.

***Hierarchical linear modeling*:** Third, as the main aim of the present study, we estimated the model parameters across the studies. In the preceding step, we used the model probabilities of each participant to create an average model for each participant and the respective session. Now, we aimed to quantify the connectivity parameters across all the sessions, participants, and studies.

To assess these group effects, we constructed HLMs using the R (R version 3.6.2, (R Core Team, 2020)) packages lme4 (lme4_1.1) and nlme (nlme_3.1) (Bates et al., 2015; Lindstrom and Bates, 1990). We decided to use hierarchical modeling instead of simple multiple linear modeling to account for the hierarchical structure in the data. Hierarchical structures were introduced by studies C and D, in which participants were measured multiple times.

The present HLM approach evaluates the magnitude of each connectivity parameter between regions. These parameters were nested into studies and further nested into repeated measurements per participant. For HLM, we deliberately used the point estimate of the posterior parameter of each participant and session after BMA.

To describe the magnitude of a particular connectivity parameter, we modeled it as a function of the study and hormone as fixed effects, respectively. Fixed effects are unknown, constant parameters, which are like regression coefficients in multiple regression analysis. We modeled the particular participant as a random effect. Random-effects represent random (unobserved) variables (West et al., 2014) instead of simple regression coefficients. More illustratively, we modeled each participant having a random intercept. Consequently, the participants’ intercepts deviated around the fixed-effect, or global, intercept.

We were not interested in the interpretation of the effects of the study, participant, or hormone. We were, however, interested in the shared connectivity across the studies, participants, and sessions. Therefore, it was important to design the model such that the global intercept can be interpreted as an average parameter estimate across the studies. To achieve this interpretation, we used contrast coding or Helmert coding on the study variable and hormone variable (Sundström, 2010). In the first contrast variable (‘AvsB’), we assigned a value of +0.5 for all the observations belonging to study A and −0.5 for all the observations belonging to study B. Next, we included study C (‘ABvsC’) by contrasting studies A and B (+0.25 each) versus study C (−0.5). We continued the same way with study D (‘ABCvsD’) by assigning +0.16 for the observations of studies A, B, and C and −0.5 for the observations of study D. Similarly, we introduced a one-level Helmert coding for the hormone variable, contrasting mid-luteal vs. early follicular phase (‘MvsP’).

For each connection of each DCM matrix, we constructed a separate HLM. Of those HLMs, we emphasized the global intercept (i.e., fixed-effect intercept) of the corresponding model. When modeling the DCM parameters of the A-matrix, B-matrix ‘faces’, and C-matrix, we included all the terms. When modeling the B-matrix ‘emotions’, we dismissed the explanatory variable ‘hormone’ because study C and study D did not include emotions in their paradigms. When analyzing the B-matrix ‘fame’, we did not include ‘hormone’ or ‘study’ as we just used study D for this analysis. As an example, a particular B-matrix connectivity parameter for the effect of ‘faces’ was modeled in the following manner:

$$y_{i}=\beta_{0}+\;\beta_{1}x_{i1}+\;\beta_{2}x_{i2}+\beta_{3}x_{i3}+\gamma x_{i4}+u_{i}+\varepsilon_{i}$$

with $y_{i}$ being the DCM parameter (response variable) of participant i, $\beta_{0}$ representing the global (fixed effect) intercept, $\beta_{1}$ to $\beta_{3}$ representing the slopes of the contrasts of the study variables $x_{i1}$ to $x_{i3}$, respectively, γ being the slope of the contrast of the hormone variable $x_{i4}$ (all fixed effects). $u_{i}$ corresponds to the random effect of ‘participant,’ and $\varepsilon_{i}$ is the random error, with $\varepsilon_{i}\sim N\left(0,{\sigma_{\varepsilon }}^{2}\right)$, and $u_{i}\sim N\left(0,{\sigma_{u}}^{2}\right)$. When modeling the parameters of other matrices, such as ‘emotion’ or ‘fame,’ particular fixed-effect terms were dismissed according to the logic described above. Using contrast coding, we tested the intercept for significance, applying a Bonferroni family-wise error correction with a threshold of $\alpha_{Bonf}=\frac{\alpha }{n}=\frac{0.05}{6}$, with n as the number of tests on interregional connections per matrix (A matrix, B matrix ‘emotion,’ and B matrix ‘fame’), and α as the native false-positive threshold.

## 3 Results

The results section is structured as follows: first, we present a comparison of all the neural models using BMS separately for each study (3.1). Second, we describe the weighted parameter estimates after participant-specific BMA for all the data sets (3.2). Last, we present the HLM results showing parameter estimates across the studies (3.3). Based on this, we propose a revised model of the core face perception network.

### 3.1 Bayesian model selection

First, we conducted a BMS separately for each study. The results for study C are presented separately for both sessions (corresponding to two different phases of the participants’ menstrual cycle). Group results are not displayed for study D because of the variable number of sessions included for each participant.

**Fig. 4:**
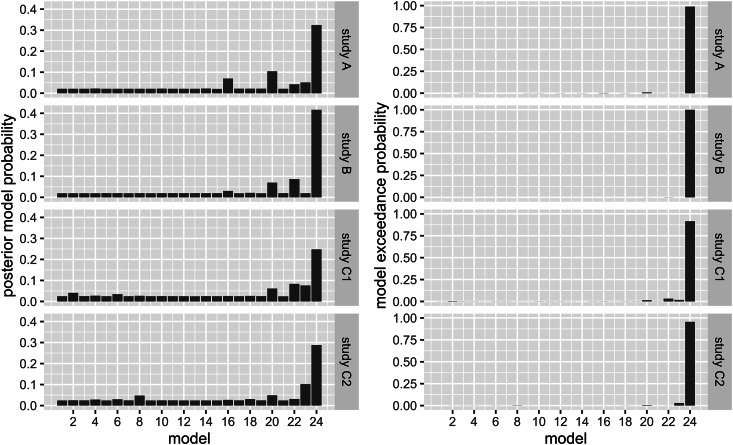
Bayesian model selection results. Left panel: The posterior model probabilities are displayed. We see that model #24 has the highest relative probability with 0.248 (study C1) to 0.417 (study B). Right panel: The model exceedance probabilities are displayed. In all the data sets, model #24 exhibited a high exceedance probability (>0.9).

The posterior probability for model #24 (see Fig. 3) was the highest in all the studies (Fig. 4, left panel), ranging from 0.248 (study C1) to 0.417 (study B). Similarly, the exceedance probabilities for model #24 — the probabilities that model #24 is more likely than any of the other models — ranged from 0.915 (study C1) to >0.999 (study B, Fig. 4, right panel). The winning model expressed the highest possible interconnectivity in each analyzed data set. In all the data sets analyzed, we discerned the same winning model with a high posterior and exceedance probability (Fig. 4). Interestingly, our winning model differs from that of study FI (see Fig. 3).

### 3.2 Bayesian model averaging

In the second step, we calculated an average model for each participant and study using BMA. BMA uses the posterior model probabilities of all the models of a particular participant and calculates a weighted average model. The weights were determined by the respective posterior model probabilities. BMA, therefore, accounts for the uncertainty of each model, as revealed by BMS (Stephan et al., 2010). Kernel density estimates of the participant-specific connectivity parameters after BMA, grouped by the respective study for the A-matrix, C-matrix, and all B-matrices, are illustrated in the supplementary results (Figs. S1–S4). The kernel density plots visualize the variability of the single participant parameter estimates grouped by the respective study.

**Fig. 5:**
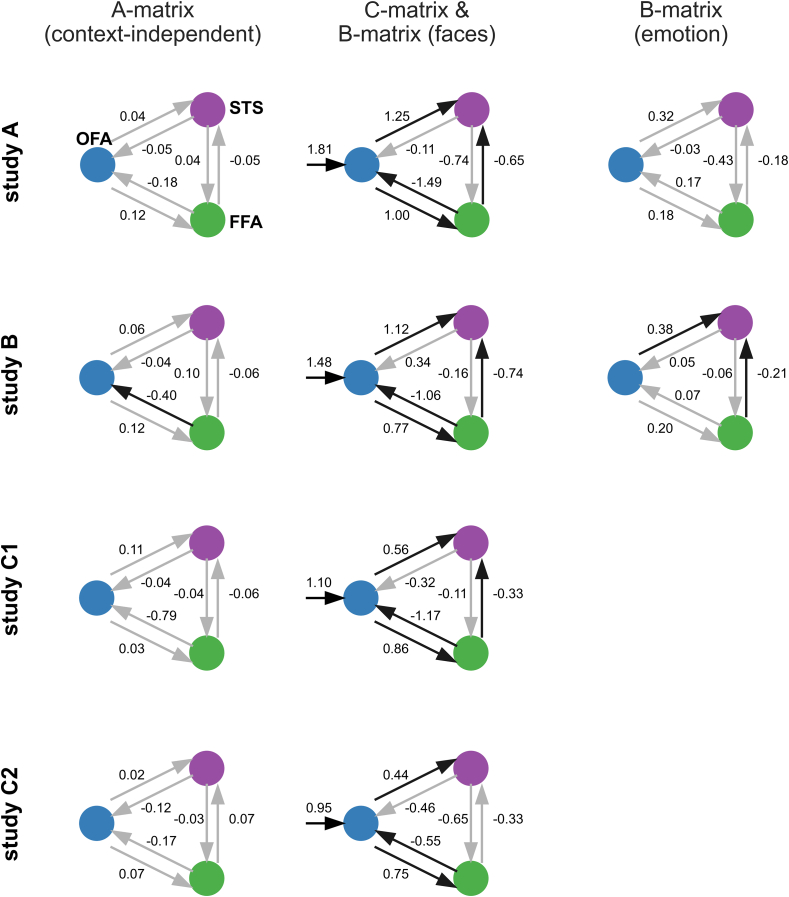
The average connectivity within each study. Studies A, B (upper panels), and C (lower panels) were divided into two scanning sessions. The connectivity between the following three regions is illustrated: the OFA (blue), FFA (green), and STS (purple). In the left panel, the A-matrix (context-independent coupling) is shown. In the middle panel, the driving input (‘faces,’ C-matrix) and B-matrix (‘faces’) are displayed, and in the right panel, the B-matrix (‘emotions’) is shown. Black arrows indicate significant connections (i.e., significant within-study). Gray arrows indicate non-significant connections. The number alongside each arrow indicates the average connection strength. Self-connections (A-matrix) were omitted in the figures but distributed around −0.5 (see Fig. S2). (For interpretation of the references to color in this figure legend, the reader is referred to the Web version of this article.)

To calculate a separate model for each study, we applied a one-sample *t*-test onto each connectivity parameter separately for each study. We used a Bonferroni-corrected threshold of p = 0.05 per matrix and study (see Methods). The average models for each study are displayed in Fig. 5. The connectivity patterns for each study were similar; although the average connections may have differed in magnitude, they tended to point in the same direction (i.e., positive or negative). Moreover, some connections exceeded the threshold for significance in one study but not in the others. Therefore, naively contemplating each study in the absence of the others could lead one to draw similar conclusions regarding many parameters while disregarding other parameters due to significance thresholds.

As a general pattern, the following was observed: within the A-matrix, the parameters were relatively small and rarely significant. The C-matrix was always significantly positive. Within the B-matrix (‘faces’), forward connections from the OFA to the FFA and the OFA to the STS were always significantly positive. Most of the time, the backward connections from the FFA to the OFA and the STS to the OFA were negative (sometimes significantly). Collateral and backward connections between the FFA and STS were always negative (sometimes significantly). The B-matrices (‘emotion’) showed weaker parameters which were rarely significant.

We tested for statistical significance across the studies in the following step to identify the global effects using HLM.

### 3.3 Hierarchical linear modeling

**Fig. 6:**
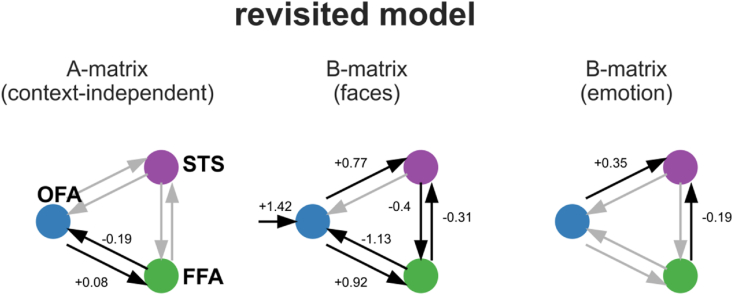
A revisited model for the core system of face perception. Driving input (‘faces’) enters the OFA. Significant connections, as revealed by HLM, are displayed with black arrows and depicted with numbers. Non-significant (determined by HLM) but present (determined by BMS) connections are illustrated by gray arrows without numbers. The context-independent connections and modulatory effects of ‘faces’ and ‘emotion’ are displayed separately. ‘Fame’ did not significantly modulate any present connection and is therefore not shown. The final model of the original study is depicted in Fig. 1 for comparison.

In the final step, we assessed the commonalities in the participant- and session-specific connectivity parameters across the studies to investigate the modulatory influences of ‘faces,’ ‘emotion,’ and ‘fame’ on the network, as well as the interregional, context-independent connection of the A-matrix and the driving input (C-matrix). We used HLM as a tool to quantify the magnitude and significance of each connection. We included all the significant connections in a new and revised model of the core face perception network (Fig. 6).

Using HLM, we identified the intercepts representing the ‘average effects across studies’ that significantly differed from zero. We have displayed all the connections in pseudo-colors in Fig. S5. Furthermore, we have displayed all the significant connections in a model-like structure in Fig. 6. First, in the context-independent connections (A-matrix), only the forward connection from the OFA to the FFA showed significant positivity (+0.08, p = 0.0016). The corresponding backward connection from the FFA to the OFA was significantly negative (−0.19, p = 3.3*10^−8^). Further, the driving input into the system (C-matrix) had a positive value (+1.42, p = 9.3*10^−31^). ‘Faces’ positively modulated the forward connection from the OFA to the FFA by +0.92 (p = 1.3*10^−17^), and that from the OFA to the STS by +0.77 (p = 2.8*10^−13^). ‘Faces’ negatively modulated the backward connection from the FFA to the OFA by −1.13 (p = 3*10^−14^), and the collateral connections from the FFA to the STS by −0.31 (p = 0.002) and vice versa by −0.4 (p = 0.0007). Similarly, ‘emotions’ positively modulated the forward connection from the OFA to the STS by +0.35 (p = 7.7*10^−6^) and negatively modulated the collateral connection from the FFA to the STS by −0.19 (p = 0.0005). However, ‘fame’ did not significantly modulate any connection.

Our resulting model has some similarities and differences compared with the original study. The similarities include the increase of forward-coupling induced by ‘faces.’ Differences mainly relate to the connections not included in the winning model of study FI. ‘Emotions’ modulated the forward connection to the STS instead of those to the FFA. We discuss possible reasons for the differences between our results and those of study FI below.

## 4 Discussion

In this study, we conducted a conceptual replication of an early network model of face perception using multiple data sets. While we successfully reproduced some aspects of the original model, the revised model was distinct in terms of some other major aspects.

We will first describe our revisited model in terms of single interactions and compare it to the original model (4.1). Secondly, we will discuss the modifications applied to our analysis pipeline compared to that of the original study (4.2). Some of these modifications were introduced by us to remedy issues in the original study, which may have limited its interpretability. Other modifications were merely due to developments within the DCM framework which have been introduced in new software versions. Further, we embed the presented network model within the broader framework of the predictive coding theory and outline some limitations (4.3). Finally, we emphasize the importance of conceptual replications in network neuroscience (4.4).

### 4.1 The revisited model of face perception

We tested face perception models consisting of the OFA, FFA, and STS, with the OFA serving as a hierarchically early input region that propagates information to the FFA and STS. As stated previously, inference in DCM is possible at different levels; it is possible at the level of the model space (i.e., which model is most likely) and parameter space (i.e., the shape of model parameters) (Stephan et al., 2010). Regarding the model space, we showed that our winning model was fully interconnected. This total interconnectivity was revealed by BMS in all the different samples and paradigms (Fig. 4); it comprised forward, backward, and lateral connections. The model proposed by study FI comprised merely forward connections (Fig. 1). Recently published studies have proposed hemispheric differences in the degree of interconnectivity. For instance, Wang et al. (2020) quantified structural, functional, and effective connectivity within the core- and extended systems of face perception. They reported higher interconnectivity within the face perception system of the right hemisphere comprising both feed-forward and feedback connections, while the left hemisphere showed a predominantly feed-forward pattern (Wang et al., 2020).

Regarding the parameter space: in all the models, the external input was modeled via the effect of ‘faces’ in the C-matrix. ‘Faces’ entered the system via the OFA according to the Haxby model. However, concurring theories propose the FFA as the input region (Rossion, 2008). As a working model, we stick to the OFA as a hierarchically earliest region and, therefore, target region for the driving experimental input, consistent with the Haxby model (Haxby et al., 2000). We further modeled the ‘effect of faces’ on every interregional connection (B-matrix). Across the studies and participants, we found five significant modulations of ‘faces’ on interregional connections. ‘Faces’ positively increased the forward connectivity from the OFA to the FFA and from the OFA to the STS; this supports the prevailing opinion that face perception drives such forward connectivity, as proposed in the original Haxby model (Fan et al., 2020; Haxby et al., 2000). Further, we found a significantly negative backward connectivity from the FFA to the OFA and collateral connectivity between the FFA and STS.

*‘*Emotion’ further increased the positive forward connection strength from the OFA to the STS and the negative coupling from the FFA to the STS in the revised model (Fig. 6). Here, we differed from the original model ((Fairhall and Ishai, 2007), Fig. 1), which proposed a positive forward modulation by ‘emotion’ from the OFA to the FFA; based on the single parameters across the studies, we could not clearly discern this across the presently analyzed paradigms. However, previous fMRI studies emphasize the importance of the STS in emotion recognition (Duchaine and Yovel, 2015; Engell and Haxby, 2007; Haxby et al., 2000; Hildesheim et al., 2020; Sliwinska et al., 2020).

Lastly, the effect of ‘fame’ was not significant, even though it was only modeled in one of our paradigms. According to lesion studies and imaging studies, face familiarity may be processed in more anterior regions, such as the anterior temporal face area in combination with the FFA (Busigny et al., 2014; Evans et al., 1995; Sergent et al., 1992; Williams et al., 2006). To disentangle the effects of ‘fame,’ models with anterior temporal face regions included might provide better insights.

### 4.2 Methodological adjustments to the original model

We deliberately introduced some modifications to the original DCM pipeline as described below.

#### 4.2.1 A-matrix and experimental effects

The A-matrix represents the context-independent coupling between regions, i.e., the underlying effective connectivity throughout the entire experiment (control conditions, fixations, etc.). Other effects, such as the effect of ‘faces’ on a particular connection, specified via the B-matrix, are additive to the context-independent parameters. Deciding which experimental effects to model in which matrices are important in the DCM workflow. We decided to model the effect of ‘faces’ explicitly in a B-matrix; this allowed us to differentiate the connectivity induced by ‘faces’ from the residual connectivity at rest or induced by any control condition. In study FI, the effect of ‘faces’ was not modeled explicitly in a B-matrix (unlike how they modeled the effects of ‘emotions’ and ‘fame’ in a B-matrix). Instead, the A-matrix parameters were interpreted as the effect of ‘faces,’ which were confounded by all the conditions present in the respective experimental runs.

Irrespective of the matrix in which study FI and our study modeled ‘faces,’ the effect of ‘faces’ highly overlapped between both studies; the positive forward connectivity from the OFA to the FFA and the OFA to the STS was present in both the original model (Fig. 1) and our revised model (Fig. 6). Backward connections were modeled in the original study but did not survive the model selection (Fairhall and Ishai, 2007).

#### 4.2.2 One-vs. two-step model selection

The effects of ‘emotion’ and ‘fame’ in the original study were modeled in a two-step approach. First, the authors assessed the coarse structure of the model by conducting a BMS that only specified the A- and C-matrices. However, it is unclear if the experimental input was properly distributed across the regions without the specification of a B-matrix onto the connections. Due to the control conditions and rest periods, the resulting A-matrix parameters potentially underestimated the true effect of ‘faces.’ Similarly, the parameters of the A-matrix were provided with more narrow shrinkage priors, much tighter than those of the B-matrix (Zeidman et al., 2019a), which under Bayesian assumptions lead to a weaker posterior parameter estimate.

However, model #2 was selected by BMS in study FI (Fig. 1, left or Fig. 3). Then, the authors added B-matrices for ‘emotion’ or ‘fame’ in the appropriate paradigms and reported the significance of the resulting coupling parameters; however, the model selection procedure did not account for these additional regressors. Therefore, the model selection could have yielded different results if these regressors had been included. For this reason, we included all the regressors (A-, B-, and C-matrices in the respective paradigms) from the beginning to avoid biasing the model selection.

#### 4.2.3 The use of different information criteria

Since the original study was published (Fairhall and Ishai, 2007), the DCM framework has undergone significant developments. One implementation was free energy (Friston et al., 2007; Penny, 2012), which became the preferred choice of information criterion. However, in study FI, Akaike information criterion (AIC) and Bayesian information criterion (BIC) were the current standard information criteria that, under certain signal-to-noise ratio conditions, are not sensitive for fully interconnected models. Instead, they deploy a high penalty for the number of parameters (i.e., model complexity) (Penny, 2012). Conversely, free energy incorporates the covariance between the parameters, increasing the sensitivity for fully connected models (Penny, 2012). However, it has also been shown that free energy overemphasizes fully connected models (Litvak et al., 2019). We additionally repeated the BMS analysis with AIC and BIC rather than F. The results are illustrated in Fig. S6 in the supplementary material and demonstrate that the different information criteria have strongly contributed to the differences in the structure of the winning model (Fig. S6). However, none of the BMS results corresponded to the results of the original study FI (Fig. S6).

#### 4.2.4 Modeling across different data sets

We included four different data sets in our analysis; thus, we needed to include covariates to control for specific independent variables of the different studies. A relatively novel method to include covariates in DCM is the parametric empirical Bayes (PEB) framework (Friston et al., 2016; Zeidman et al., 2019b). This framework allows second-level dynamic causal models to assess the effects of covariates across a group or between groups. However, using PEB was not practical in our study, as we dealt with different dependencies and B-matrices for each data set. Further, within the PEB framework, participants are weighted differently according to their respective model fit; we wanted each participant to be weighted rather equally in a group analysis. Due to these reasons, we decided to use HLM instead of PEB.

### 4.3 Face perception revisited in the predictive coding framework

In the following, we embed our resulting main model parameters (Fig. 6, Fig. S5) into the broader context of predictive coding as the predictive coding framework generally seems well-suited for such hierarchical models. Despite the oversimplification of the complex predictive coding theory, we integrated our model in the predictive coding framework for a comprehensive and meaningful interpretation at the level of the resulting parameter estimates.

Briefly, in the predictive coding framework, the brain is organized into hierarchical interconnected modules. Each module communicates predictions (i.e., expectations about its input) to the respective lower level. Similarly, each module calculates a prediction error as the discrepancy between the prediction (i.e., the expected signal from the lower level) and input (i.e., received a signal from the lower level). The prediction error is then propagated to the respective higher level, wherein the prediction is updated. The updated prediction is then propagated back to the respective lower level (prediction updating). This iterative process is described on a microscopic scale (Bastos et al., 2012) within the early visual hierarchy (Rao and Ballard, 1999) and on a macroscopic scale in the context of DCM (Chen et al., 2009; Den Ouden et al., 2009).

In this framework, we might interpret the positive parameters from lower regions (OFA) to hierarchically higher regions (FFA and STS) as prediction error signaling, analogous to a forward propagation of the signal along the hierarchy (Fig. 6). Conversely, we might interpret the negative backward connections from higher to lower level areas as prediction updating. Prediction updating in Bayesian networks is equivalent to “explaining away the stimulus” (Gotts et al., 2012), whereby the causes of the sensory input are learned, and the prediction error, which is the neural activation that results in the positive forward-coupling, gets reduced. It is plausible that over the course of an experimental simulation, the presence of a particular input stimulus (either a sequence of faces or a single face, depending on the experimental paradigm) is learned, therefore “explained away,” causing the positive and negative couplings on a macroscopic scale.

In the previous paragraphs, we deliberately detailed an interpretation of the positive forward connectivity and negative backward connectivity in the context of the predictive coding theory. However, positive forward connectivity appears to be the most obvious option available. If all three neural regions are activated by faces within the respective fMRI paradigms, and the input regressor of faces (C-matrix) enters the system via the OFA, the obvious explanation in the context of the full model (#24 in Fig. 3) is a positive forward transfer to the other two regions. Alternatively, a positive forward connection to one region and positive collateral connectivity from this region to the remaining regions could also be an alternative pathway to activate all the regions. Analogous effects may be the easiest way to explain the positive activity by face perception within all three regions in the context of other models, such as those evolving from prototypes 1 and 3 (Fig. 3).

The lack of alternatives for the general expression of the parameters can also be seen in the negative backward connectivity by ‘faces’ from the FFA to the OFA. Usually, negative self-connections within a region induce a decrease in activity within that region over time (e.g., during the whole modeled experiment) and prevent the system from becoming epileptic. However, self-connections in our models were context-independent, as they were only present in the A-matrix, and we did not allow the modulation of those in the B-matrix. Therefore, the inhibitory parameters remained the same throughout the course of the experiment, regardless of whether it was an experimental or control condition. In the experimental condition (‘faces’), the activity in all the presently modeled regions was higher (see the definition of the regions for the extraction of the time series). Therefore, allowing only the connections from other regions (instead of self-connections) to downregulate this additional activity may have caused such a manifestation of negative couplings between regions. This concerns the negative backward couplings from the FFA and STS toward the OFA, which downregulate the OFA activity. Further, this concerns the negative collateral connections between the FFA and STS, which downregulate the STS and FFA, respectively.

Experiments and simulations are required to validate these theories in the future. However, such effects, implicitly introduced by the setup of the models, limit any extensive interpretation of our revised model or any similar model. However, complementary imaging techniques such as EEG/MEG, which have a far better temporal resolution, might shed light on the time-sensitive orchestrations between the regions during bottom-up and top-down processing. For instance, a recent study by Fan et al. (2020) investigated response times of the regions of the core system using specialized paradigms to untie top-down and bottom-up processes within the predictive coding framework (Fan et al., 2020). Interpretations using fMRI however can rather be made for long-lasting interactions in the brain.

### 4.4 The requirement of conceptual replications

As we have already discussed in the introduction, neuroimaging findings are often vulnerable to non-replication (Gorgolewski and Poldrack, 2016). DCM may be particularly vulnerable to this due to the massive number of degrees of freedom a researcher is faced within the analysis. Additionally, changes in the experimental setup, pipeline, statistical methods, and even software versions can cause significant changes in the parameter estimates (Bedenbender et al., 2011; Botvinik-Nezer et al., 2020; Frässle et al., 2016b; Weissenbacher et al., 2009). As we can usually only investigate very narrow hypotheses in a single study, we highly depend on the validity and reproducibility of the previous results being built upon. Therefore, we need more conceptual replications and meta-analyses of models like that in the present study. Most importantly, we need to be critical and mindful while interpreting previously published results.

## 5 Conclusion

The aim of our study was to conceptually replicate the main findings of Fairhall and Ishai (2007) on the effective connectivity within the core system of face perception. Across four different data sets, we demonstrated that our revised model was more complex than the originally proposed model, with a high degree of interaction between regions.

## Funding

This work was supported by funds from the 10.13039/501100001659Deutsche Forschungsgemeinschaft (DFG, grant no. JA 1890/11-1) and from the 10.13039/100009103von-Behring-Röntgen Stiftung (grant no. 63-0030).

## Data and material availability

Data of study D is available online (https://www.openneuro.org, accession number ds000117, (Wakeman and Henson, 2015)). First-level analyses from study A are available at github.com/kesslerr/efp. From all the studies, data is fully available for the DCM modeling, beginning with the DCM models, model comparison results, model averaging results, hierarchical linear modeling pipeline, and other statistical tests at github.com/kesslerr/coreSysRev. Further data can be provided on request.

## Declarations of competing interest

None.
