# Supplementary material for: Revisiting the effective connectivity within the distributed cortical network for face perception
Source: Neuroimage Rep. 2021 Aug 17;1(4):100045. doi: 10.1016/j.ynirp.2021.100045 (PMC12172785; doi:10.1016/j.ynirp.2021.100045)
Supplement: Multimedia component 1 [file mmc1.docx]

## Supplementary Material

### Supplementary Methods

#### Paradigms

##### Paradigm A

##### To investigate brain connectivity during the observation of different basic emotions, a paradigm was designed to present image sequences of neutral, happy, angry, or fearful faces as well as houses in a block design. The face stimuli (39 individuals) were taken from the Radboud Face Database (RaFD, (Langner et al., 2010)), whereas the house stimuli were freely available pictures taken from the internet. The stimuli were transformed into gray-scale images and cropped to 500*400px. Furthermore, they were matched in mean luminance using the SHINE toolbox for MATLAB (Willenbockel et al., 2010). To avoid lateralization effects due to low-level image properties like asymmetries in the faces or houses, each image was mirrored vertically in one half of its appearances. A fixation cross was shown in the center of each stimulus, as well as during the inter-stimulus-intervals and inter-block-intervals in the center of the screen. The participants were advised to maintain the fixation of their gaze during the entire experiment. Each of the five conditions appeared 20 times, resulting in a total of 100 blocks. Within each block, a sequence of 24 images was shown. A face or house stimulus was shown for 350 ms followed by a fixation cross for 150 ms, resulting in a total block length of 11.85 s (main article, Fig. 2). A jittered inter-block-interval of approximately 3.3–7.3 seconds was introduced to reduce anticipation effects. Additionally, four pause-trials of 25 seconds were included, appearing after the 21st, 40th, 60th, and 80th stimulus block, in which the participants were instructed to relax and close their eyes. To ensure the attention of the participants during the stimulus presentation, a 1-back task was introduced. The participants were instructed to press a button with the index finger of both hands whenever a stimulus was shown twice in a row, which happened 1–3 times in each block. The total duration of the experiment was approximately 30 minutes.

##### Paradigm B

##### To investigate the connectivity between the core system of face perception and the amygdala during the observation of fearful faces, stimuli showing neutral faces, fearful faces, or houses were presented in a block design. The face stimuli (30 individuals) were taken from the Karolinska Directed Emotional Faces dataset (<http://www.emotionlab.se/resources/kdef>) (Lundqvist et al., 1998), whereas the house stimuli were freely available pictures taken from the internet. The stimuli were transformed into gray-scale images and cropped to 600*530px. Furthermore, they were matched in mean luminance using the SHINE toolbox for MATLAB (Willenbockel et al., 2010). The participants were advised to fixate their gaze on the nasion of the faces. For the house stimuli, they were asked to maintain their eyes at about the same height as that of the fixation for the face stimuli. Each of the three conditions (neutral faces, fearful faces, and houses) was repeated 14 times, resulting in a total of 42 blocks. Within each block, a sequence of 20 images was shown. The face or house stimulus was shown for ~310 ms followed by a fixation cross for ~390 ms, resulting in a total block length of 14.5 s. We used an inter-block-interval of ∼6.5 s. Additionally, a pause-trial of 30 s in which the participants were instructed to relax and eventually close their eyes for a moment was included after half of the stimuli were presented. To ensure the attention of the participants during stimulus presentation, a 1-back task was introduced. The participants were instructed to press a button with the index finger of both hands whenever a stimulus was shown twice in a row, which happened 1–3 times in each block.

##### Paradigm C

The participants simply viewed blocks of faces, houses, and scrambled pictures. The pictures of houses and faces were obtained from a standardized database. The scrambled pictures were generated using a Fourier transformation. In total, 14 blocks of every condition appeared in a randomized order, including a 20 s break after half of the experiment. Every block contained 20 stimuli (faces, houses, or scrambled pictures), which were presented for 300 ms in the middle of the screen. Between each block, a fixation cross was shown for 12 s. The stimuli were controlled for brightness and contrast and presented in different grey scales. The participants were asked to push a button on a response box as soon as they saw the same stimuli in immediate succession. Successive stimuli were presented 3–4 times in every condition. The total length of the paradigm was 13 min.

##### Paradigm D

See (Wakeman and Henson, 2015) for further information on this paradigm.

##### Paradigm FI

The authors conducted five experimental runs within one session, scanning 10 participants (Fairhall and Ishai, 2007). They presented line drawings of unfamiliar faces (1 run) and gray-scale photographs of unfamiliar (two runs), famous (one run), and emotional faces (fearful and happy, one run). Each image was presented for 3 seconds. As a visual baseline, scrambled versions of the stimuli were used. The stimuli were presented in a block fashion, with a duration of 36 s for the experimental condition and 24 s for the control condition. The experimental and control conditions were each presented thrice per run.

#### Data acquisition

##### Data set A

The MRI data were acquired using a 3.0-Tesla MR scanner (Siemens TIM Trio, Erlangen, Germany) with a 12-channel head matrix receive coil at the Core Unit Brainimaging, Department of Psychiatry and Psychotherapy, University of Marburg. A high-resolution structural data set was acquired using a T1-weighted magnetization-prepared-rapid gradient-echo sequence with the following parameters: acquisition time, 4 min 18 s; repetition time (TR), 1900 ms; echo time (TE), 2.52 ms; field of view, 256 mm; matrix, 256x246; slice thickness (ST), 1 mm; phase encoding direction (PE), anterior » posterior; distance factor (DF), 50 %; flip angle, 9°; parallel imaging generalized autocalibrating partially
parallel acquisitions with acceleration factor, 2; bandwidth, 170 Hz/Px; sagittal, ascending acquisition; 176 slices.

Functional images were collected using a T2*-weighted gradient-echo echo-planar imaging sequence (EPI) sensitive to the blood oxygen level-dependent (BOLD) contrast. TR, 1550 ms; TE, 36 ms; matrix, 72x72; phase oversampling, 12 %; ST, 2.7 mm; DF, 15 %; voxel size, 2.8x2.8x2.7 mm (2.8x2.8x3.1 mm incl. gap); PE, anterior » posterior; flip angle, 70 °; bandwidth, 1654 Hz/Px; no parallel imaging; ascending acquisition; 20 slices with the measurement volume aligned to the anterior-posterior commissural line. The volume covered the whole temporal and occipital lobes, and the inferior frontal gyrus.

##### Data set B

Functional images were collected using a T2*-weighted gradient-echo echo-planar imaging sequence (EPI) sensitive to the BOLD contrast. The parameters were as follows: TR, 1610 ms; TE, 36 ms; matrix, 96 x 128; ST, 2.4 mm; DF, 15 %; voxel size, 2.0x2.0x2.4 mm; PE, anterior » posterior; flip angle, 70 °; bandwidth, 1346 Hz/Px; partial fourier, 7/8; no parallel imaging; ascending acquisition; 18 slices with the measurement volume aligned to the most ventral parts of the temporal and occipital poles. The slab covered the whole temporal and occipital lobes.

##### Data set C

All MRI data were acquired using a 3-Tesla TIM-Trio MR Scanner (Siemens Medical Systems) at the Department of Psychiatry and Psychotherapy, Philipps-University, Marburg. High resolution T1-weighted anatomical images were acquired from every participant (TE, 2.26 ms; TR, 1.9 ms; flip angle, 9°; matrix, 256 x 256; 176 sagittal slices; ST, 1 mm). To minimize head movements, the participants’ heads were fixated with foam pads. Functional images were collected with a T2* weighted EPI sequence sensitive to the BOLD contrast (matrix, 64x64; field of view, 192 mm; 30 slices [descending]; ST, 4 mm [15% gap]; TR, 1450ms; TE, 25ms; flip angle, 90°). Slices covered the whole brain and were positioned in a transaxial parallel direction to the anterior-posterior commissural line. The BOLD responses to different experiments were recorded with this sequence. A total of 565 scans were recorded for the face perception task. The initial four images were excluded. Contrary to data set A and B, the measurement volume covered the whole cortex.

##### Data set D

See (Wakeman and Henson, 2015) for further information on this paradigm.

#### Functional imaging data analysis

##### Data set A

All the fMRI data sets were analyzed using the Statistical Parametric Mapping software (SPM12, release 6685, Welcome Department of Cognitive Neurology, Institute of Neurology, London, United Kingdom) based on MATLAB (version 8.3 R2014a). The initial three functional images were excluded from further analysis due to T1 stabilization effects, as implemented in the protocol of the MR scanner system. The field maps were calculated using the acquired phase and magnitude images from a field map sequence. These were converted to voxel displacement maps to unwarp geometrically distorted EPI images. In a combined realignment and unwarping step, the effects of static and movement-related susceptibility-induced distortions were corrected for, as well as within-participant motion correction through a rigid body (six parameters) spatial transformation. Each participant’s functional images were also normalized to the Montreal Neurological Institute space. For an accurate transformation, each participant's T1-weighted image (coregistered to the mean functional image) was segmented, bias-corrected, and spatially normalized using the segmentation algorithm as implemented in SPM12 (formerly called "New Segment" in SPM8). The resulting forward deformation field was used for registering the realigned functional images to the Montreal Neurological Institute space that were subsequently resampled to a resolution of 2 x 2 x 2 mm^3^ and blurred with an isotropic Gaussian filter of 6mm full width at half maximum.

Statistical analyses were performed within a general linear model framework to create a 3-dimensional map in relation to the estimated regressor response amplitude. At the single-participant level, the task was modeled in a block design with BOLD responses for each condition (neutral, happy, angry, and fearful faces as well as houses, respectively) convolved with the canonical hemodynamic response function. Inter-block intervals and breaks were not modeled. The six realignment parameters of the motion correction procedure were included in the statistical model as nuisance regressors to correct for residual head movement. For each participant, differences in brain activation between the ‘face’ and control conditions were calculated. High pass filtering was applied with a cut-off frequency of 1/256 Hz to attenuate low-frequency components. At the group level, the weighted ß-images (‘face’ conditions vs. control condition) were entered into one-sample t-tests.

##### Data set B

The preprocessing and statistical analysis of data set B were performed in the same way as those for data set A, but using MATLAB version 7.8 R2009a and a high-pass filter of 1/128 Hz.

##### Data set C

All the fMRI data were analyzed with the software package SPM 8 (v4290) ([www.fil.ion.ucl.ac.uk/spm](http://www.fil.ion.ucl.ac.uk/spm)) using standard routines and templates running on MATLAB 7.7.0.471 (R2008b) (The MathWorks, Inc.). SPM 8 was used for realignment, normalization, smoothing, and statistical analysis. The functional images were realigned, normalized to a resulting voxel size of 2 x 2 x 2 mm^3^, smoothed with a 5-mm isotropic Gaussian filter, and high-pass filtered by a cut-off period of 128 s. After preprocessing, the statistical analysis was performed. BOLD responses for the test and control conditions were modeled by a boxcar function convolved with the canonical hemodynamic response function employed by SPM8. Parameter estimates (β-) and t-statistic images were calculated, describing the activation differences between the test and control conditions. For the face perception tasks, three conditions were modeled (faces, houses, scrambled images; the instruction was not modeled). Additionally, the six realignment parameters were included as covariates in each design matrix. At the group level, the weighted ß-images were entered into one-sample t-tests. The “2* faces > (houses + scrambled)” contrast, describing the differences in the patterns of brain activation between the activation and control conditions, was calculated for each participant.

##### Data set D

See (Wakeman and Henson, 2015) for further information on the processing steps.

##### Data set FI

See (Fairhall and Ishai, 2007) for further information on the processing steps.

### Supplementary Results

#### Single participant BMA parameters

Here, we describe the distribution of the single participant BMA parameters. For the A-matrix (Fig. S1, left panel), we found relatively small parameter estimates ranging from approximately -1 to +1. Most distributions appeared roughly symmetrical and centered around zero. For the C-matrix (Fig. S1, right panel), the parameters were mostly positive and ranged from -0.058 to +6.626. Positive values are expected here, as we extracted the time series for the DCM construction from the voxels that exceeded significance in the contrasts (e.g., ‘faces’ conditions vs. ‘non-face’ conditions, depending on the respective study). Therefore, in these voxels, the activation should be higher during the ‘faces’ conditions, which is consequently a positive input. The connectivity parameters for the ‘face’ condition (B-matrix, Fig. S3) were spread more widely, i.e., from approximately -4 to +4. The distributions of a parameter over participants indicated that some parameters shifted more into the positive or negative range, respectively. Connectivity parameters for the ‘emotions’ and ‘fame’ conditions (B-matrices, Fig. S3) seemed to be more symmetrically accumulated around zero compared to those for the ‘faces’ conditions.


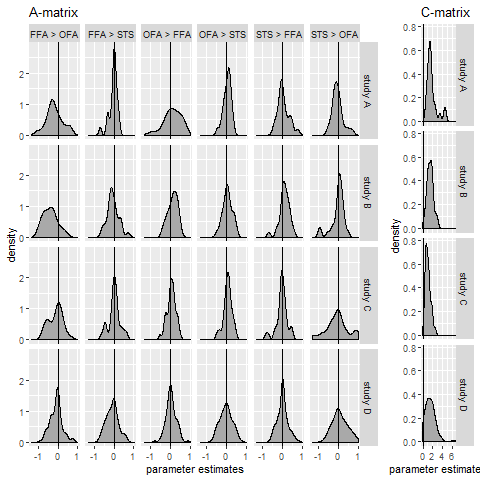


Fig. S1*:* Kernel density estimates of all the connectivity parameters of the interregional (i.e., off-diagonal) connections of the A-matrix (left panel) and C-matrix (right panel) after participant-specific BMA.


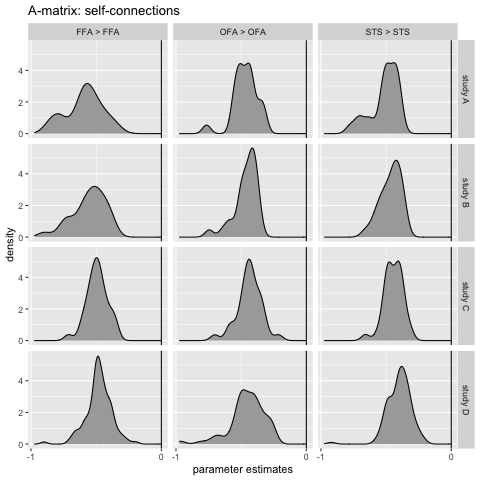


Fig. S2*:* Kernel density estimates of all the connectivity parameters of the self-connections (i.e., on-diagonals) of the A-matrix after participant-specific BMA.


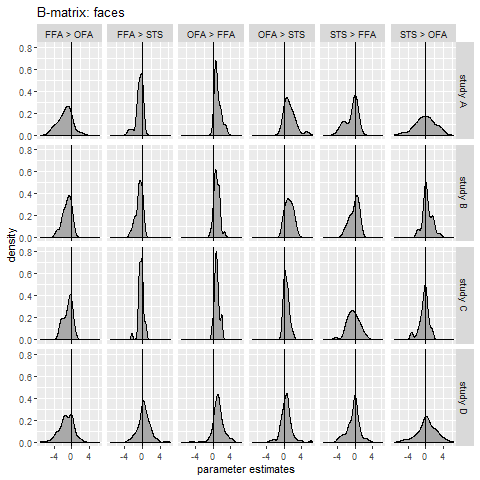


Fig. S3: Kernel density estimates of all the connectivity parameters of the B-matrix ‘faces’ after participant-specific BMA.


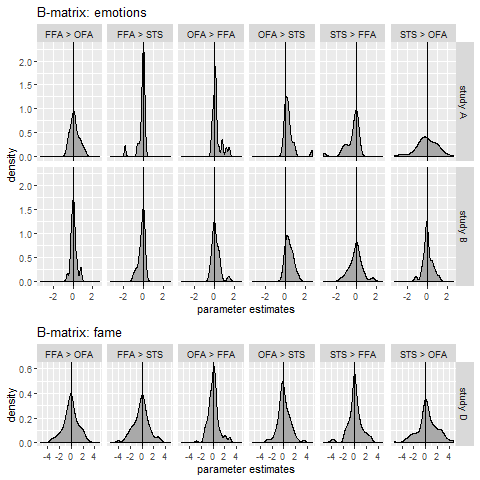


Fig. S4: Kernel density estimates of all the connectivity parameters of the B-matrix *‘*emotions’ (top panel) and *‘*fame’ (lower panel) after participant-specific BMA. Note the different scaling of the axes for the regressors ‘emotions’ and ‘fame’.

#### Hierarchical linear modeling results


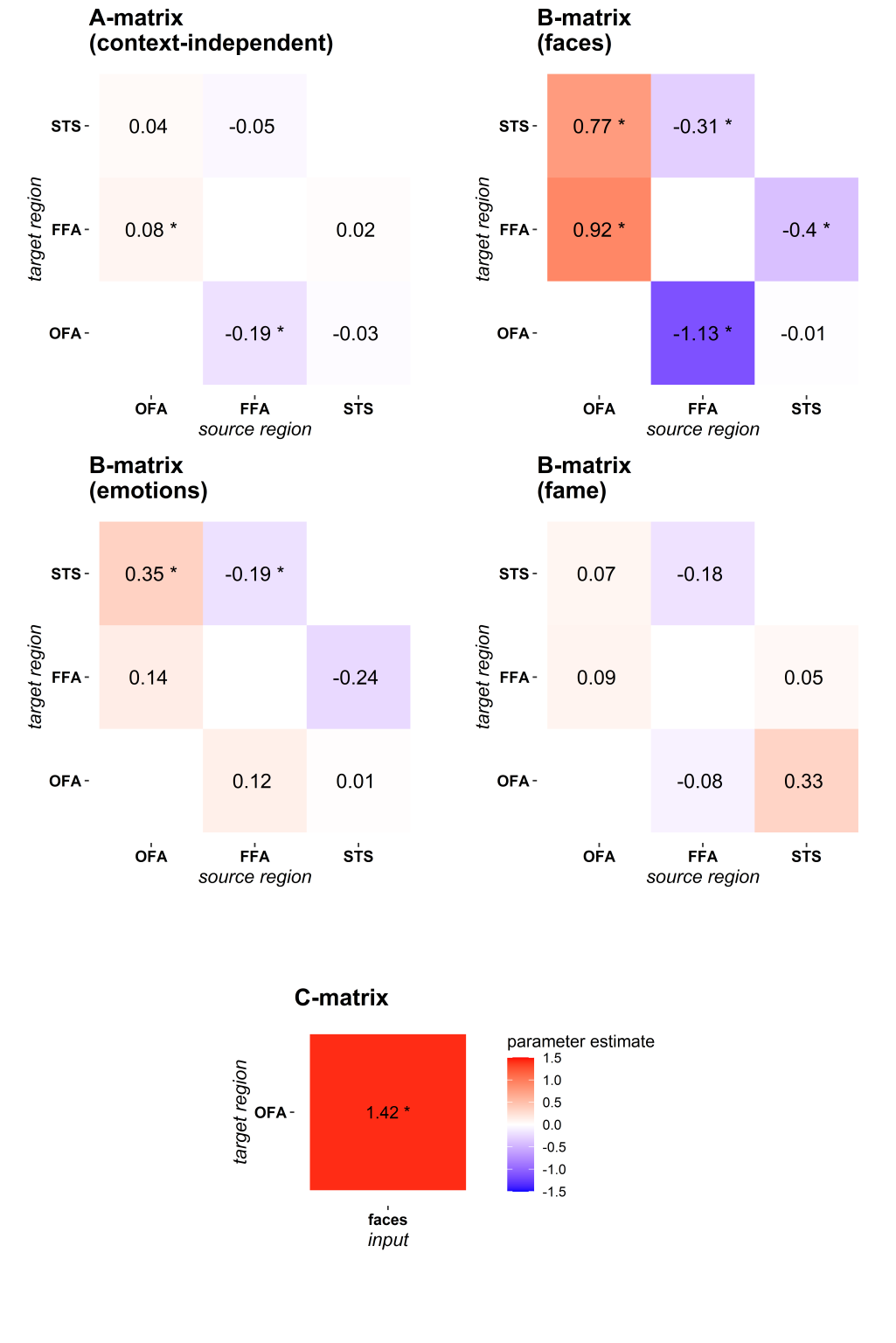


Fig. S5*:* Connectivity parameters across studies as revealed by HLM. For each matrix (A-, B-‘faces’-, B-‘emotions’, B-‘fame’, and C-matrix), the connection strength is displayed using pseudo colors ranging from -1.5 (blue) to +1.5 (red), as indicated by the color bar at the bottom right and the values in each cell. Significance is indicated by additional asterisks. The source and target regions are labeled on the x- and y-axis of each matrix, respectively. For the C-matrix, there is no source region, but the ‘faces’ regressor targets the OFA. The significant parameters of this figure are further displayed in a model-like manner in Fig. 6.

#### Alternative approximations of the log model evidence

Since the publication of SPM8, free energy (F) is the preferred choice as an approximation to the log model evidence. Before (i.e., up to SPM5), Akaike information criterion (AIC) and Bayesian information criterion (BIC) have been largely used for this purpose. The authors of study FI used both criteria as well (Fairhall and Ishai, 2007). In our study using the free energy criterion, model #24 showed striking superiority to the competing models throughout all examined data sets (Fig. 3, Fig. 4). Study FI, which applied the AIC and BIC, found that a sparser model was superior to the competing models (Fig. 3, Fairhall and Ishai, 2007). Some of the advantages and possible disadvantages of the free energy criterion have been outlined in Section 4.2. We hypothesized that the choice of the free energy criterion may largely drive differences in posterior model probabilities, and therefore the winning models between study FI and our study. To test this, we repeated the BMS for study A, B, and the two sessions of study C (C1 and C2) by applying AIC, BIC, and F separately. The results are illustrated in Fig. S6. Using AIC, either model #22 or model #24 have the highest exceedance probabilities, depending on the data set analyzed. Model #22 misses one connection compared to the full model #24, the unidirectional connection from FFA to STS (the corresponding opposite connection is however present, Fig. 3). Using BIC, either model #5, model #6, model #22, or model #24 have the highest exceedance probabilities. Model #6 does not express backward connections from FFA to OFA and from STS to OFA. Model #5 further misses the unidirectional connection from STS to FFA (the corresponding opposite connection is however present, Fig. 3). Altogether the BMS was highly variable across data sets when using AIC/BIC. Most strikingly, none of the single BMS results corresponded to the BMS result of study FI, which found model #2 to have the highest posterior probability.


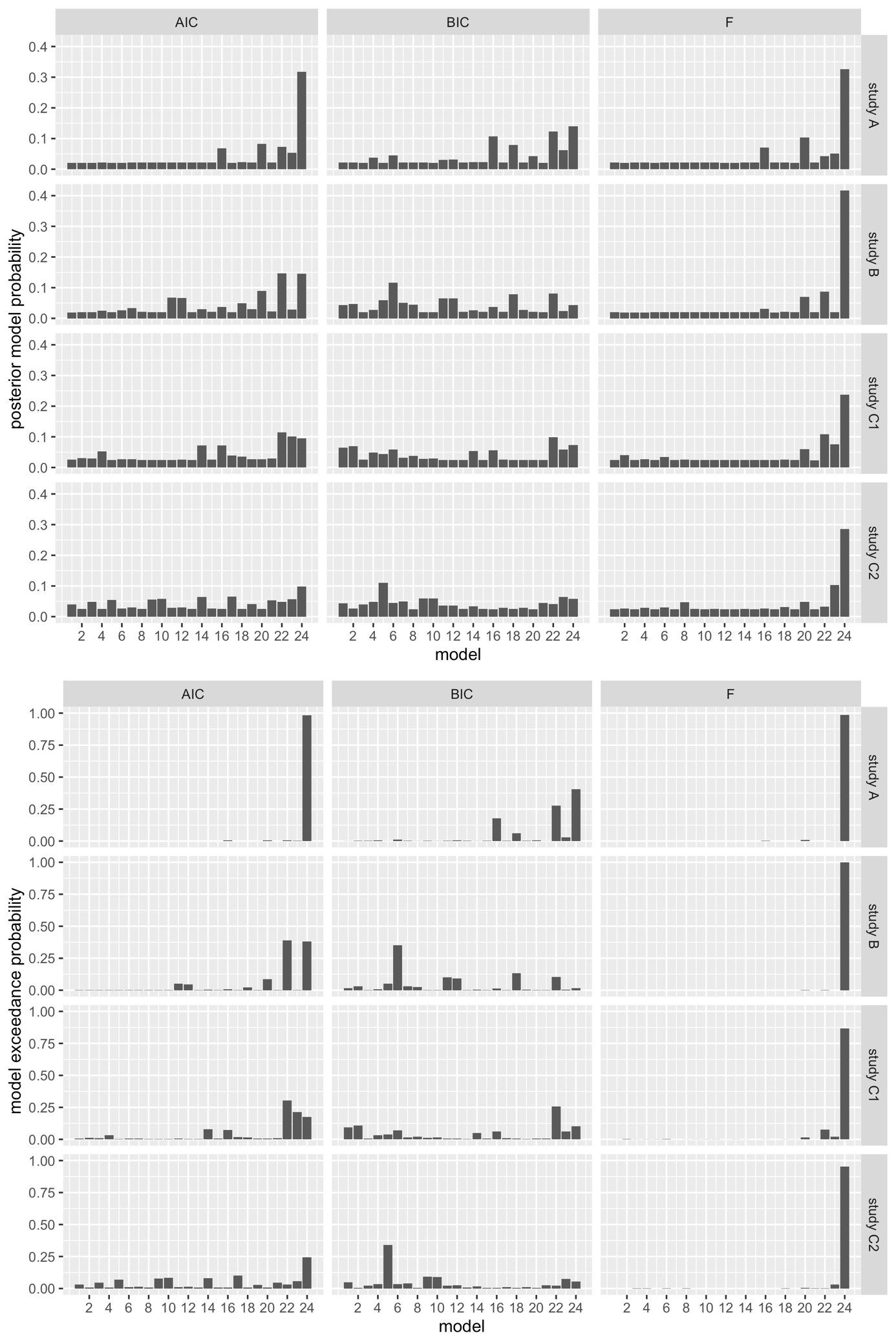


Fig. S6: BMS results using different approximations for log model evidence. Upper panel: posterior model probabilities. Lower panel: Model exceedance probabilities. The different data sets (A, B, C1, and C2) for which a BMS was possible are separated along the vertical axes. The BMS results of these studies are separated for AIC, BIC, and F as information criteria along the horizontal axis. The panels for F correspond to the results displayed in Fig. 4.

Fairhall, S.L., Ishai, A., 2007. Effective connectivity within the distributed cortical network for face perception. Cereb. Cortex 17, 2400–2406. https://doi.org/10.1093/cercor/bhl148

Langner, O., Dotsch, R., Bijlstra, G., Wigboldus, D.H.J., Hawk, S.T., van Knippenberg, A., 2010. Presentation and validation of the radboud faces database. Cogn. Emot. 24, 1377–1388. https://doi.org/10.1080/02699930903485076

Lundqvist, D., Flykt, A., Ohman, A., 1998. The Karolinska directed emotional faces (KDEF). CD ROM from Dep. Clin. Neurosci. Psychol. Sect. Karolinska Institutet.

Wakeman, D.G., Henson, R.N., 2015. A multi-subject, multi-modal human neuroimaging dataset. Sci. data 2, 150001. https://doi.org/10.1038/sdata.2015.1

Willenbockel, V., Sadr, J., Fiset, D., Horne, G., Gosselin, F., Tanaka, J., 2010. The SHINE toolbox for controlling low-level image properties. J. Vis. https://doi.org/10.1167/10.7.653
